# Supplementary material for: Diamond mirrors for high-power continuous-wave lasers
Source: Nat Commun. 2022 May 11;13:2610. doi: 10.1038/s41467-022-30335-2 (PMC9095672; doi:10.1038/s41467-022-30335-2)
Supplement: Supplementary file 1 — Supplementary Information [file 41467_2022_30335_MOESM1_ESM.pdf]

Supplementary information for “Diamond mirrors for high-power continuous-wave lasers”

Supplementary Discussion 1: finite difference time domain design and simulations.

A global optimization is performed to design a mirror that has maximum reflectivity and spectral bandwidth while also considering fabrication limitations (see Methods). Simulations are performed using a commercial finite difference time domain (FDTD) solver (Lumerical). In all simulations, periodic boundary conditions are applied in the *x*- and *y*- directions with a plane wave source as the input to a mirror. Schematic of the “golf tee” column that comprises a mirror is depicted in Fig. 1C, with all relevant dimensions labeled.

First we simulate the optical properties of a mirror for varying column dimensions. Supplementary Fig. 1 shows the reflection spectrum of a diamond mirror for varying radius r_{disc}, with all other column dimensions indicated in the figure caption, illustrating the large range of operating wavelengths of our design. It is important to note that r_{disc} (hence, the operating wavelength of a mirror) is controlled by the precision of the electron beam lithography tool, in which nanometer accuracy is attainable. Supplementary Fig. 2 depicts the reflection spectrum for varying radius r_{min}, again with all other dimensions indicated in the figure caption. For r_{min} < 75 nm the reflection spectrum is unchanged, illustrating the device’s robustness to uncertainty in etch rate or depth. The optical mode begins to leak into the support structure and substrate for r_{min} > 75 nm, abruptly reducing the reflectivity of a diamond mirror. Supplementary Figs. 3 and 4 show the reflection spectrum for varying radius $r_{support}$ and column height *h*, respectively, again with all other dimensions in the caption of each figure, with the reflection spectrum maintained for $r_{support}$ < 300 nm, and *h* > 3 µm, respectively, further indicating tolerance of our design against varying etch depth. The reflection spectrum for varying pitch, i.e. the center-to-center distance between each column, is shown in Supplementary Fig. 5, with all other dimensions indicated in the caption. We find results which parallel that of Supplementary Fig. 1, in that the target wavelength of a mirror may be varied over a large range by changing the pitch, and importantly, can also be set with a precision that is determined by the electron beam tool. Overall, our simulations indicate that the properties of a mirror can be broadly tuned, benefitting electron beam precision, but with tolerance to variations in etch depth, which is harder to control during fabrication.

Further FDTD modeling is carried out to probe the resonant nature of a diamond mirror. Supplementary Fig. 6A depicts the reflection spectrum of Fig. 1D with a dashed white line at a design angle α = 75°. See the caption of Fig. 1D for the remaining column design parameters of the mirror. The reflection spectrum for α = 75° is plotted in Supplementary Fig. 6B, and was chosen because it displays two distinct reflection maxima, corresponding to electric- or magnetic-dipole resonances. The insets of Supplementary Fig. 6B illustrate the electric and magnetic field amplitude profiles in the *x*-*y* plane of the column at *z* = 0 µm, coordinate origin is indicated in Fig. 1D. Plots of the electric and magnetic field (direction and magnitude) vectors at these resonances are shown in Supplementary Fig. 6C, with the column surface at *z* = 0 µm indicated by the blue circle. The electric field is orthogonal to the magnetic field as expected, further confirming the existence of clear electric and magnetic dipole resonances in each column.

Maps of the standing-wave patterns that are reflected from a diamond mirror are also generated with FDTD modelling. Supplementary Figs. 6D and 6E illustrate the reflected electric field amplitudes at the aforementioned electric and magnetic resonances of the diamond mirror and compares them

to the field maps obtained from a perfect electric conductor (PEC) mirror[1]. Supplementary Fig. 6D shows the standing wave patterns for the PEC mirror and the diamond mirror at the electric dipole resonance. The phase of the reflection relative to the input is close to 180°, consistent with the phase of the reflection that is obtained from a PEC (the electric field amplitude at the PEC-air boundary is always pinned to zero). Supplementary Fig. 6E illustrates the standing wave patterns for the PEC mirror and the diamond mirror at the magnetic dipole resonance. The phase of the reflection remains close to zero, consistent with the properties of a magnetic mirror. The wave patterns in Supplementary Figs. 6D and 6E are normalized to the electric or magnetic field amplitudes at their dipole resonances, respectively, clearly indicating the phase shift of the standing wave patterns generated by reflections of the diamond mirror.

References

1. Liu, S. et al. Optical magnetic mirrors without metals. *Optica* **1**, 250-256 (2014).

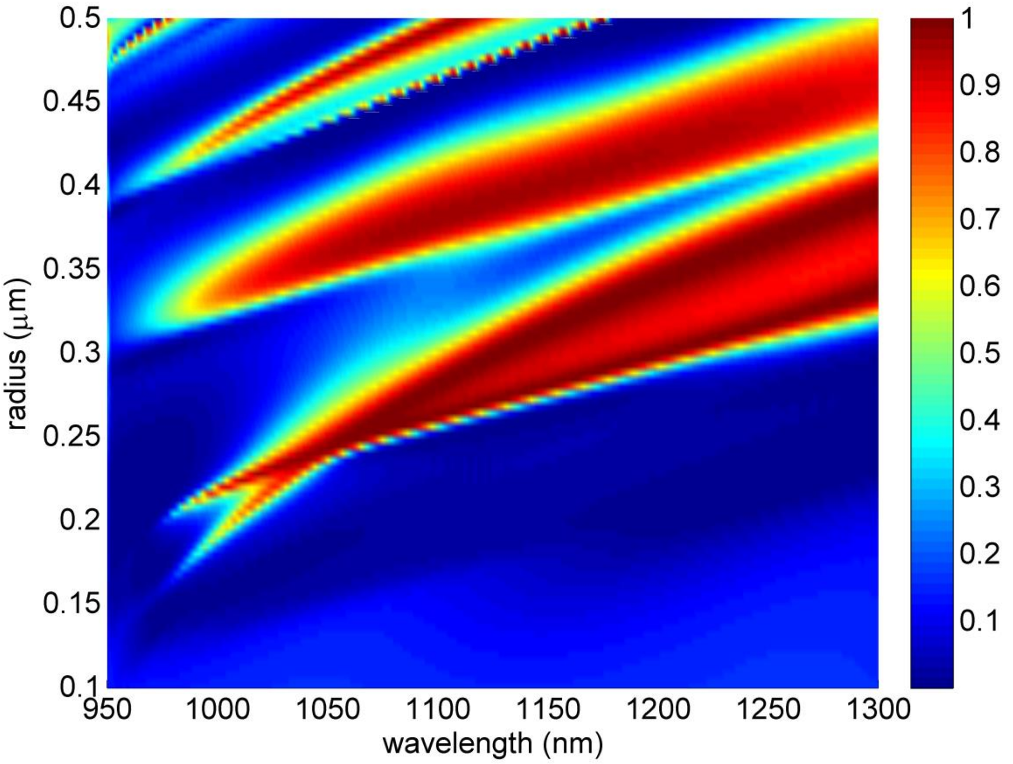

Supplementary Fig. 1 | Reflection spectrum with varying radius r_{disc}. Diamond mirror reflection spectrum at normal incidence for varying radius r_{disc}. Design parameters are: angle α = 70°, r_{min} = 50 nm, $r_{support}$ = 250 nm, pitch 1.1 μm, and h = 3 μm. Red and blue colours indicate locations of maximum and minimum reflection, respectively.

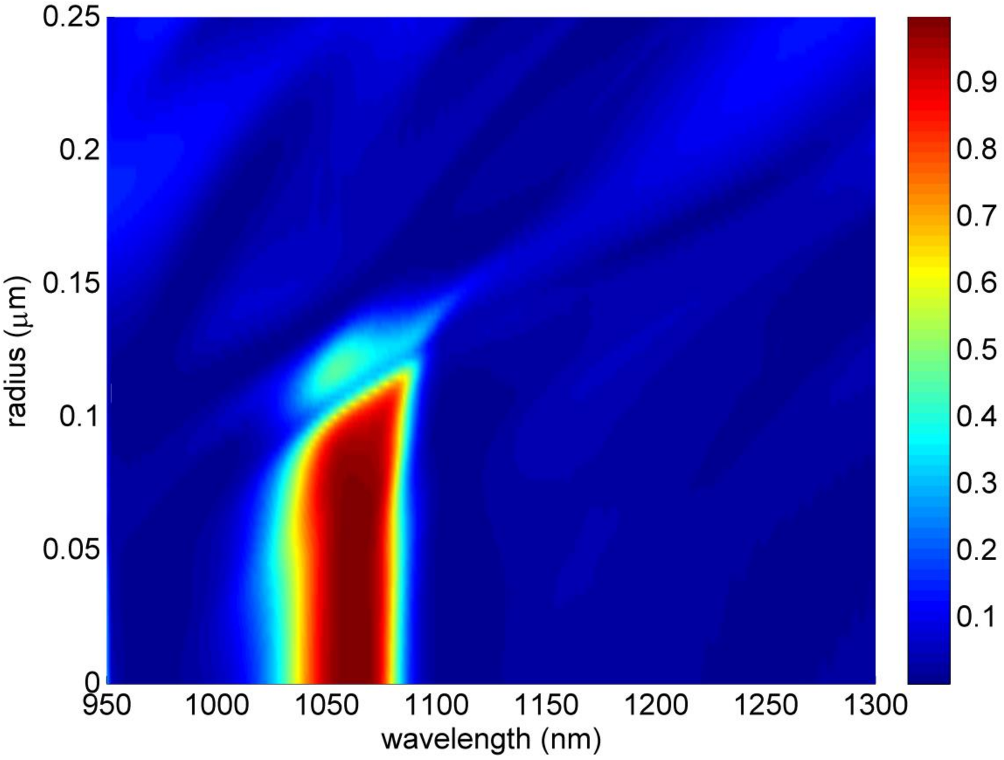

Supplementary Fig. 2 | Reflection spectrum with varying radius r_{min}. Diamond mirror reflection spectrum at normal incidence for varying radius r_{min}. Here, α = 70°, r_{disc} = 250 nm, $r_{support}$ = 250 nm, pitch 1.1 µm, and h = 3 µm. Red and blue colours indicate positions of maximum and minimum reflection respectively.

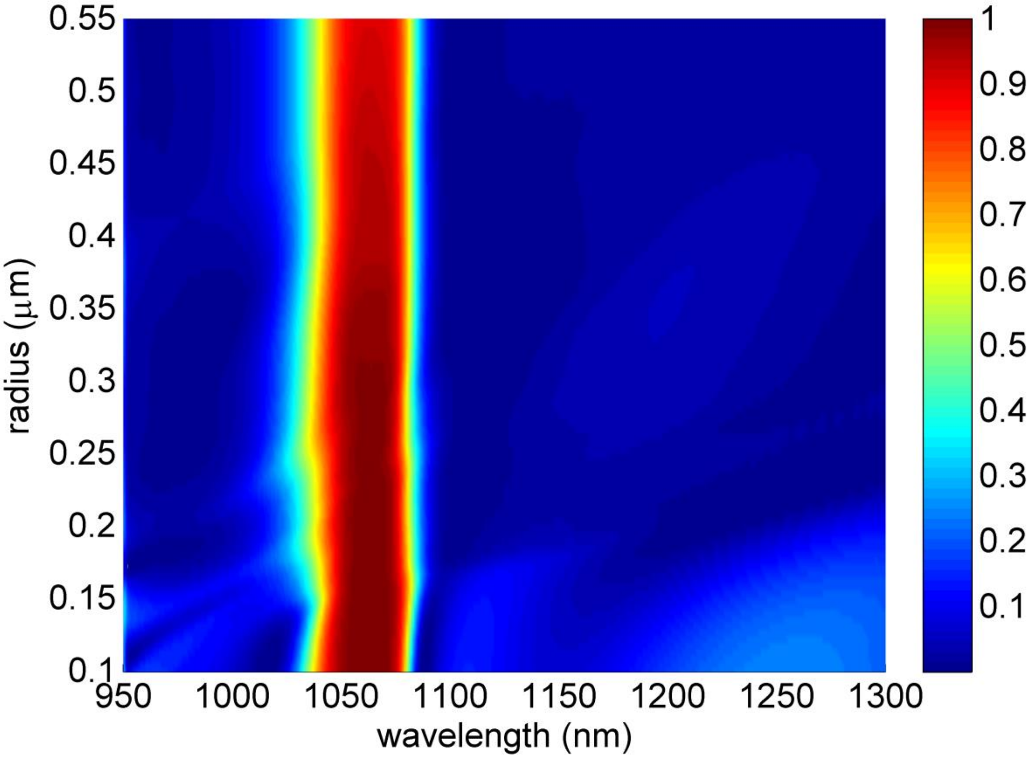

Supplementary Fig. 3 | Reflection spectrum with varying radius $r_{support}$. Diamond mirror reflection spectrum at normal incidence for varying radius $r_{support}$. Here, α = 70°, r_{disc} = 250 nm, r_{min} = 50 nm, pitch 1.1 µm, and h = 3 µm. Red and blue colours indicate positions of maximum and minimum reflection respectively.

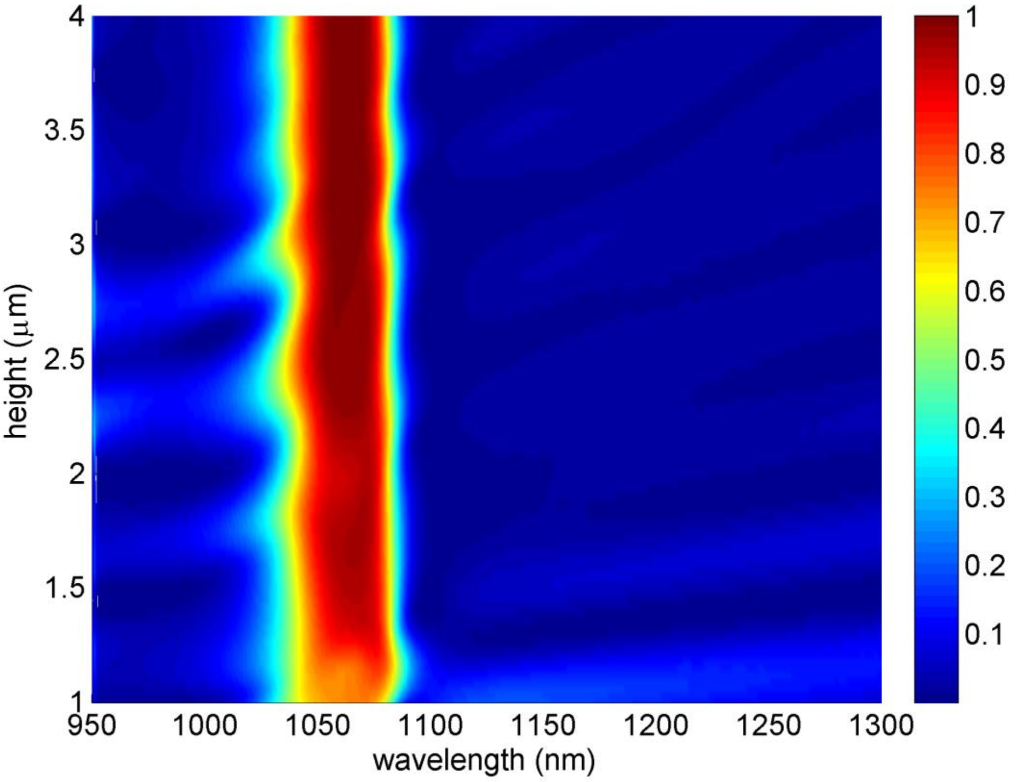

Supplementary Fig. 4 | Reflection spectrum with varying total device height *h*. Diamond mirror reflection spectrum at normal incidence for varying height *h*. Here, α = 70°, r_{disc} = 250 nm, r_{min} = 50 nm, radius $r_{support}$ = 250 nm and pitch 1.1 µm. Red and blue colours indicate positions of maximum and minimum reflection respectively.

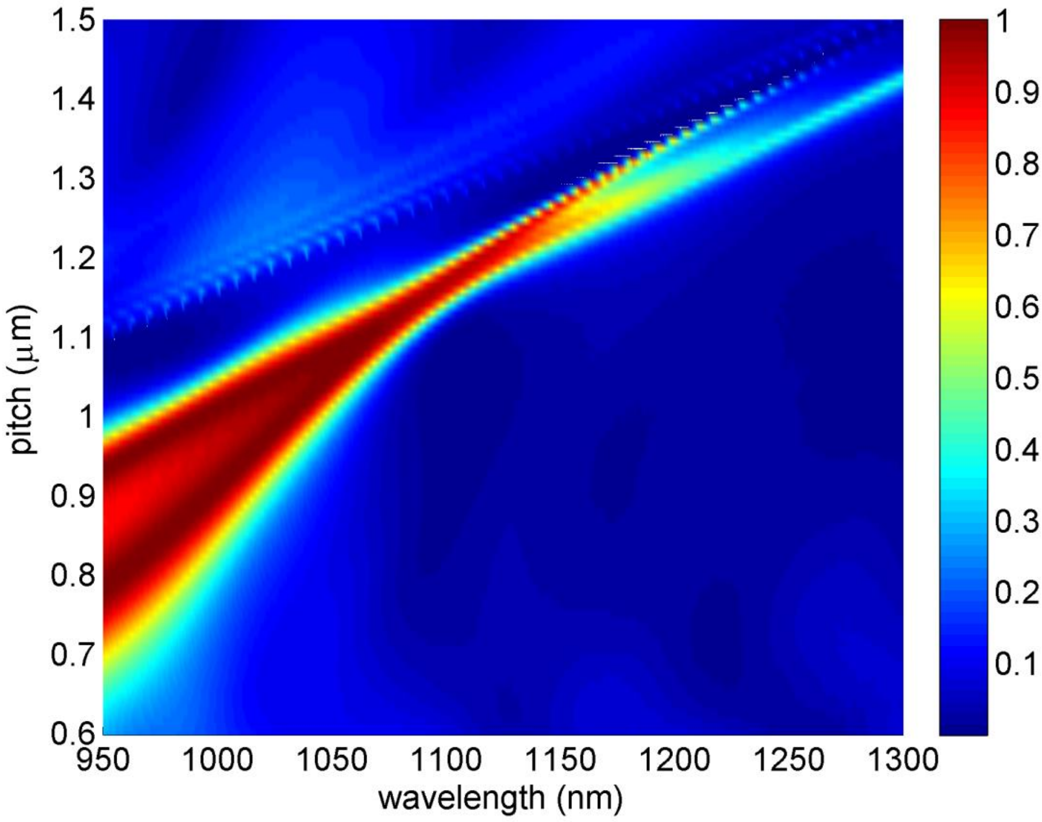

Supplementary Fig. 5 | Reflection spectrum with varying pitch. Diamond mirror reflection spectrum at normal incidence for varying pitch. Pitch is defined as the center-to-center distance between each of the columns that are arranged in a hexagonal lattice. Here, α = 70°, r_{disc} = 250 nm, r_{min} = 50 nm, r_{support} = 250 nm and h = 3 µm. Red and blue colours indicate positions of maximum and minimum reflection respectively.

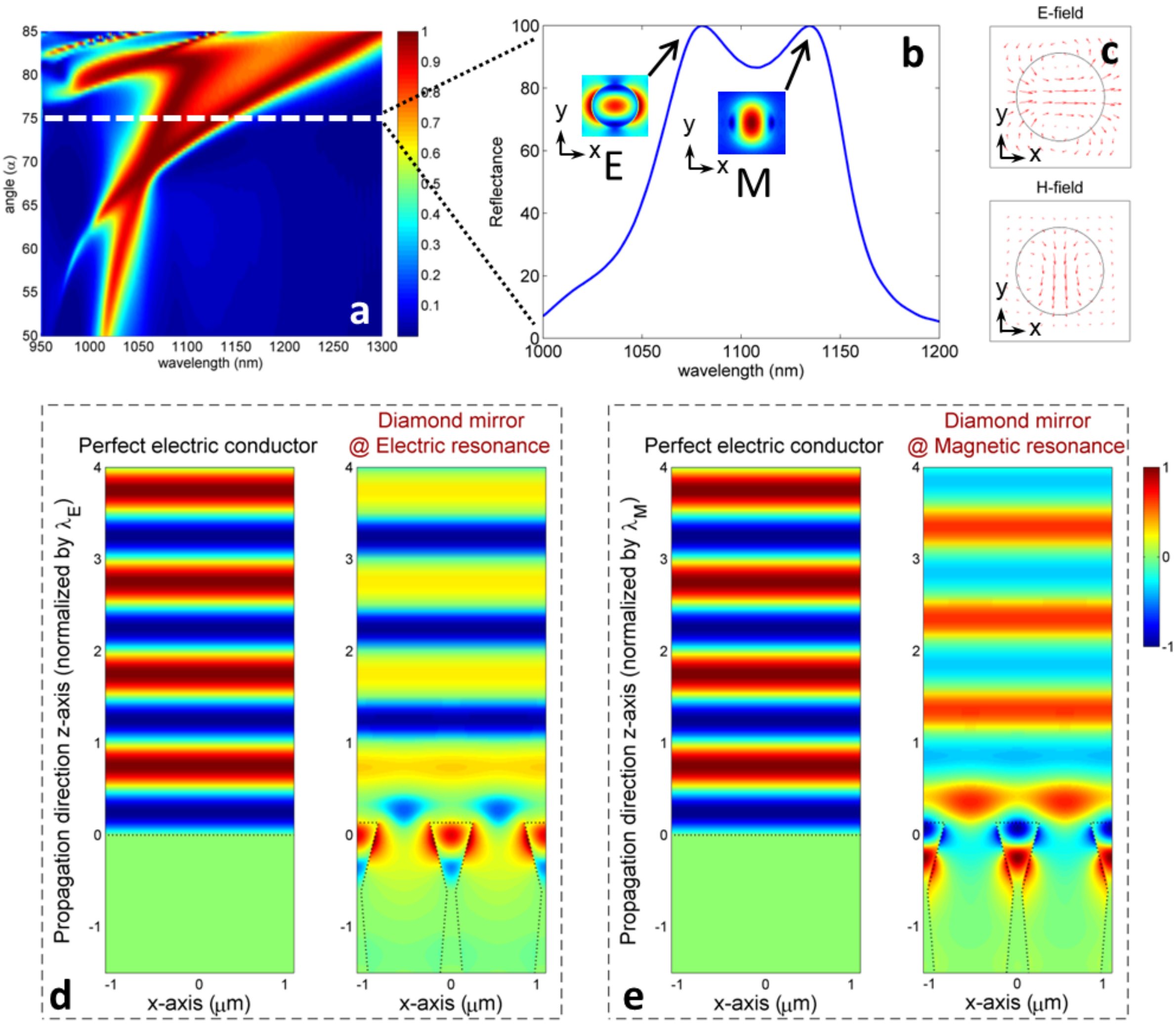

Supplementary Fig. 6 | Standing-wave patterns reflected from a diamond mirror and a perfect electric conductor (PEC) mirror. a, Reflection spectrum of a diamond mirror, with a sectional line-cut at α = 75° indicated by the white dashed line. Red and blue colours indicate locations of maximum and minimum reflection, respectively. **b,** Perfect reflection exists for both spectrally separated electric and magnetic modes. Insets show the electric and magnetic field plots in the *x*-*y* plane of the golf tee column. Colours indicate the electric field amplitude. **c,** Electric and magnetic field vector plots confirm the presence of electric and magnetic dipolar resonances in the golf tee column. **E**-field and **H**-field vectors are perpendicular to each other. **d,** Standing-wave pattern of reflected light for the diamond mirror at the electric resonance compared to the reflection from PEC. 180° phase shift in the reflected light, characteristic for reflection from PEC, can be observed. The dotted black lines represent the PEC-air interface and the boundaries of the diamond nanostructures. The PEC surface is placed at a height equivalent to the center of the mode in the golf tee column. Colours indicate the electric field amplitude. **e,** In contrast, the diamond mirror excited at the magnetic resonance features a zero phase shift, as seen in standing wave pattern. Colours indicate the electric field amplitude.
