# Supplementary material for: Diamond mirrors for high-power continuous-wave lasers
Source: Nat Commun. 2022 May 11;13:2610. doi: 10.1038/s41467-022-30335-2 (PMC9095672; doi:10.1038/s41467-022-30335-2)
Supplement: Supplementary file 3 — Description of Additional Supplementary Information [file 41467_2022_30335_MOESM3_ESM.pdf]

Description of Additional Supplementary Information

Supplementary Video 1.

Thermal video capture of the LIDT test on a diamond mirror using a λ=1070 nm continuous wave laser, focused to a 750 µm ($1/e^2$) diameter spot using 500 W of power, exposure for 30 s. Video speed at 0.5x to highlight the thermal evolution. The hot spot is directly below the number "1" label.

Supplementary Video 2.

Thermal video capture of the LIDT test on a diamond mirror using a λ=1070 nm continuous wave laser, focused to a 750 µm ($1/e^2$) diameter spot using 2.5 kW of power, exposure for 30 s. Video speed at 0.5x to highlight the thermal evolution. The hot spot is directly below the number "1" label.

Supplementary Video 3.

Thermal video capture of the LIDT test on a diamond mirror using a λ=1070 nm continuous wave laser, focused to a 750 µm ($1/e^2$) diameter spot using 5 kW of power, exposure for 30 s. Video speed at 0.5x to highlight the thermal evolution. The hot spot is directly below the number "1" label.

Supplementary Video 4.

Thermal video capture of the LIDT test on a diamond mirror using a λ=1070 nm continuous wave laser, focused to a 750 µm ($1/e^2$) diameter spot using 7.5 kW of power, exposure for 30 s. Video speed at 0.5x to highlight the thermal evolution. The hot spot is directly below the number "1" label.

Supplementary Video 5.

Thermal video capture of the LIDT test on a diamond mirror using a λ=1070 nm continuous wave laser, focused to a 750 µm ($1/e^2$) diameter spot using 10 kW of power, exposure for 30 s. Video speed at 0.5x to highlight the thermal evolution. The hot spot is directly below the number "1" label.

Supplementary Video 6.

Video capture of the LIDT test on a diamond mirror using a λ=1070 nm continuous wave laser, focused to a 750 µm ($1/e^2$) diameter spot using 10 kW of power. Video helps illustrate the power leaking through the diamond and being absorbed by the water-cooled stage which begins to glow. The diamond is held between two copper clamps, with the etched section of the diamond (i.e. the mirror) appearing grey in colour.

Supplementary Video 7.

Thermal video capture of the LIDT test on a dielectric mirror using a λ=1070 nm continuous wave laser, focused to a 750 µm ($1/e^2$) diameter spot using 10 kW of power, exposure for 30 s. Laser damage occurs at approximately 10 s of exposure. Video speed at 0.5x to highlight the thermal evolution. The hot spot is directly below the number “1” label.
